# The ABC Method and Gastric Cancer: Evidence From Prospective Studies

**DOI:** 10.2188/jea.JE20160140

**Published:** 2016-12-05

**Authors:** Shizuka Sasazuki

**Affiliations:** Division of Prevention, Center for Public Health Sciences, National Cancer Center, Tokyo, Japan

**Keywords:** *Helicobacter pylori*, pepsinogen, gastric cancer

In this issue, Ikeda et al^1^ reported on the predictive ability of the combination of *Helicobacter pylori* (*H. pylori*) antibody and serum pepsinogen (sPG), known as the ABC method, for gastric cancer occurrence in a long-term prospective study. The Hisayama Study is known for its long follow-up period and high rates of medical examinations, autopsies, and follow-up. The most important finding in this report is that adding sPG further improved the accuracy and discriminatory ability of the risk prediction model of developing gastric cancer compared with *H. pylori* alone. Based on several evaluation indices, Ikeda et al concluded that the combination of *H. pylori* and sPG is a significant predictor for the development of gastric cancer over a long-term period.

It is also notable that the authors ultimately combined Group C (positive for *H. pylori* antibodies and sPG) and Group D (negative for *H. pylori* antibodies and positive for sPG) because there was no significant difference in the cumulative incidence of gastric cancer in these groups. Similar findings were observed in a recent meta-analysis of prospective studies in Eastern Asians^2^: compared to Group A, the summary hazard ratios for developing gastric cancer for Groups B, C, and D were 2.50 (95% confidence interval [CI], 1.24–4.51), 10.00 (95% CI, 5.20–17.51), and 15.00 (95% CI, 7.50–27.11), respectively. When compared to Group C, the summary estimate for Group D was 1.56 (95% CI, 0.84–2.65). Based on a sample of almost 20 000 individuals from the Japan Public Health Center-based prospective study (the JPHC Study), Charvat et al observed a similar pattern. The corresponding hazard ratios were 7.58 (95% CI, 4.16–13.79), 13.86 (95% CI, 7.76–24.75), and 14.09 (95% CI, 7.03–28.26) for Groups B, C, and D, respectively.^3^ Using the model developed based on the ABC method and lifestyle factors, estimates of the 10-year probability of gastric cancer occurrence ranged from 0.04% (95% CI, 0.02%–0.10%) to 14.87% (95% CI, 8.96%–24.14%) for men and from 0.03% (95% CI, 0.02%–0.07%) to 4.91% (95% CI, 2.71%–8.81%) for women.^3^

A risk prediction model is a simple and effective method for evaluating individualized risk by quantifying cancer risk. In the era of personalized medicine, prediction models are expected to play a role in screening for high-risk groups, assisting medical decision-making and health education, and so on. The combination of *H. pylori* and sPG is expected to be an effective tool for determining gastric cancer risk.^4^ The findings of Ikeda et al in this issue provide further evidence that the method can stratify middle-aged healthy adults by gastric cancer risk.

The proportion of participants categorized as Groups A, B, C, and D was 25%, 46%, 26%, and 3% in the Hisayama Study and 29%, 30%, 38%, and 3% in the JPHC Study, respectively. Although the prevalence of *H. pylori* positivity has been declining in Japan in recent years, the proportion in Groups B, C, and D might still be relatively high. Therefore, it is conceivably difficult to use the ABC method as the primary screening method in Japan. This method has been introduced in some gastric cancer primary screening settings in Japan, but how to use this method in the real world has not yet been established. These preventive measures could be used to assist in choosing subjects for gastric cancer screening (eg, when, who, and how frequently). In addition, it may provide an incentive for individuals to undergo clinical examinations earlier when they become aware of the symptoms. The next step in this research might be to clarify the role of the ABC method by aggregating data and exploring how to apply this method to the real world by monitoring existing cumulative data.
